# Comparison of long-read sequencing and MLPA combined with long-PCR sequencing of *CYP21A2* mutations in patients with 21-OHD

**DOI:** 10.3389/fgene.2024.1472516

**Published:** 2024-11-01

**Authors:** Tian Lan, Jin Wang, Kaibi Chen, Jianru Zhang, Xiaohong Chen, Hui Yao

**Affiliations:** ^1^ Department of Genetics, Metabolism and Endocrinology, Wuhan Children’s Hospital, Tongji Medical College, Huazhong University of Science and Technology, Wuhan, China; ^2^ GrandOmics Biosciences Co, Ltd., Beijing, China

**Keywords:** 21-hydroxylase deficiency (21-OHD), CYP21A2, long-read sequencing (LRS), targeted capture, multiplex ligation probe amplification (MLPA), genotypes

## Abstract

**Background:**

21-Hydroxylase deficiency (21-OHD) is caused by mutations in the *CYP21A2* gene. Due to the complex structure and the high genetic heterogeneity of the *CYP21A2* gene, genetic testing for 21-OHD is currently facing challenges. Moreover, there are no comparative studies on detecting *CYP21A2* mutations by both second-generation sequencing and long-read sequencing (LRS, also known as third-generation sequencing).

**Objective:**

To detect *CYP21A2* variations in 21-OHD patients using targeted capture with LRS method based on the PacBio (Pacific Biosciences) Sequel II platform.

**Methods:**

A total of 67 patients with 21-OHD were admitted in Wuhan Children’s Hospital. The full sequence of CYP21A2 gene was analyzed by targeted capture combined with LRS based on the PacBio Sequel II platform. The results were compared with those of long-polymerase chain reaction (Long-PCR) combined with multiplex ligation probe amplification (MLPA) detection. Based on the *in vitro* study of 21-hydroxylase activity of common mutations, the patient genotypes were divided into groups of Null, A, B, and C, from severe to mild. The correlation between different genotype groups and clinical typing was observed.

**Results:**

The study analyzed a total of 67 patients. Among them, 44 (65.67%) were males and 23 (34.33%) were females, with a male-to-female ratio of approximately 1.9:1. A total of 27 pathogenic variants were identified in the 67 patients, of which micro-conversion accounted for 61.9%, new variants of *CYP21A2* accounted for 8.2%; deletion accounted for 22.4% (*CYP21A2* single deletion and chimeric *TNXA*/*TNXB* accounted for 12.7%, chimeric *CYP21A1P*/*CYP21A2* accounted for 9.7%); and duplication accounted for 3.0% (*CYP21A2* Gene Duplication). I2G was the most common variant (26.9%). Targeted capture LRS and MLPA combined with Long-PCR detection of *CYP21A2* mutations showed 30 detection results with differences. The overall genotype-phenotype correlation was 82.1%. The positive predictive rate of the Null group for salt wasting (SW) type was 84.6%, the A group for SW type was 88.9%, the group B for simple virilization (SV) type was 82.4%, and the group C for SV type was 62.5%. The correlation coefficient r_s_ between the severity of the phenotype and the genotype group was 0.682 (*P* < 0.05).

**Conclusion:**

Targeted capture combined with LRS is an integrated approach for detecting *CYP21A2* mutations, allowing precise determination of connected sites for multiple deletions/insertions and cis/trans configurations without analyzing parental genomic samples. The overall genotype-phenotype correlation for 21-OHD is generally strong, with higher associations observed between genotype and phenotype for group Null, A, and B mutations, and larger genotype-phenotype variation in group C mutations. Targeted capture with LRS sequencing offers a new method for genetic diagnosis in 21-OHD patients.

## 1 Introduction

21-hydroxylase deficiency (21-OHD, OMIM #201910) is an autosomal recessive genetic disease, accounting for more than 95% of congenital adrenal hyperplasia (CAH) (Pignatelli et al., 2019). The root cause of the condition is the deficiency of 21-hydroxylase in the adrenal corticosteroid synthesis pathway, resulting in impaired production of cortisol and aldosterone. Based on differences in genotype and residual 21-hydroxylase activity, 21-hydroxylase deficiency (21-OHD) can be classified into classic and non-classic types (Hannah-Shmouni et al., 2017). The classic type is further divided two subtypes: the salt wasting (SW) type with almost complete loss of enzyme activity, and the simple virilization (SV) type that retains 1%–2% of enzyme activity. Non-classic 21-OHD typically presents with milder clinical symptoms.

The 21-hydroxylase (21-OH), is encoded by the *CYP21A2* gene, which is located in the main histocompatibility complex III region of chromosome 6p21.3, with a full length of 3.35 kb and composed of 10 exons (Higashi et al., 1986). The *CYP21A2* is located within the tandem repeat sequence RCCX module. The RCCX module is one of the most complex copy number variation (CNV) loci in humans, and gene misalignment may occur during meiosis, leading to gene conversion, unequal crossing, deletion, and the formation of non-functional chimeric genes (White et al., 1986). About 30 kb from the *CYP21A2* gene, there is a highly conserved pseudogene, *CYP21A1P*, which has up to 98% and 96% sequence identity with the real gene respectively (Higashi et al., 1986). The genes in this region vary significantly in size and copy number (Parajes et al., 2008). Currently, the Human Gene Mutation Database (HGMD) Professional Edition (Stenson et al., 2017) has reported nearly 500 mutations in the *CYP21A2* gene, of which 388 disease-causing mutations (DM) are associated with the 21-OHD phenotype (http://www.hgmd.cf.ac.uk/). About 75% of these are due to micro-conversion of the non-functional *CYP21A1P* gene, 20%–25% are deletions or duplications, and 1%–2% are novel *CYP21A2* variants (Hannah-Shmouni et al., 2017; Tusie-Luna and White, 1995).

At present, 21-OHD is screened by testing 17-hydroxyprogesterone (17-OHP) levels, and the false positive rate fluctuates between 0.4% and 9.3% (Auer et al., 2023; Hayashi et al., 2017). The genetic testing is the gold standard for determining the cause of CAH. Previous generation sequencing used Sanger combined with quantitative polymerase chain reaction (QPCR) to detect point mutations and deletion duplications (CNVs), with read lengths of only 600–1,000 bp. Currently, the commonly used method is next-generation sequencing (NGS), which employs Whole Exome Sequencing (WES) combined with multiplex ligation probe amplification (MLPA) detection. NGS-WES detects variations in genes that cause CAH besides *CYP21A2*. *CYP21A2* mutations are detected using long-polymerase chain reaction (Long-PCR) to obtain target gene detection point mutations. MLPA detects deletions and duplications. MLPA has higher sensitivity and better reproducibility than QPCR. However, the read length of NGS is about 200 base pairs, which can only fuzzily match the reference sequence and can not detect pseudo-genes that are highly similar to both the target region and homologous sequences. At the same time, in the process of PCR amplification in NGS sequencing, GC bias will affect the detection accuracy. NGS can’t determine specific types of chimeras caused by complex rearrangements or large deletions. LRS, also known as third-generation sequencing, can achieve read lengths ranging from 15 kb to 2 Mb for DNA sequencing, which is nearly ten-thousand-fold improvement over NGS. At the same time, the capture process can reduce GC bias, which can significantly improve the detection accuracy. A single read of LRS can span the entire *CYP21A2* gene (3.35 kb), accurately mapping to the reference genome and distinguishing it from its pseudogene *CYP21A1P*. The unique advantage of LRS in long read length enables comprehensive detection of single nucleotide variations (SNVs), deletions/insertions, tandem duplications, structural variations, differentiation between true gene and pseudogenes, methylation, and cis/trans configurations. This approach provides a new solution for detecting pathogenic variants in the *CYP21A2* gene. This study uses targeted capture and LRS techniques to sequence the genes of 67 patients with 21-OHD, to achieve a comprehensive genetic analysis of all CAH-related gene variants.

## 2 Materials and methods

### 2.1 Research object

This study included 67 patients diagnosed with 21-OHD who visited the Department of Genetic Metabolism and Endocrinology, Wuhan Children’s Hospital from January 2013 to October 2023. The diagnostic criteria refer to “*Congenital adrenal hyperplasia due to steroid 21-hydroxylase deficiency: an Endocrine Society clinical practice guideline*” (Speiser et al., 2018). The patients were divided into three groups: SW, SV, and NC. This study was approved by the Ethics Committee of Wuhan Children’s Hospital (Ethical Review Number 2023R054-E02). All legal guardians of the patients signed informed consent forms.

### 2.2 Genetic testing methods

#### 2.2.1 Long-read sequencing

Collect 2 mL of peripheral blood from the patient and send it to Beijing Grandomics Biosciences Co., Ltd. for targeted capture sequencing of CAH-related genes including *CYP21A2*, *CYP11A1*, *CYP11B1*, *CYP17A1*, *HSD3B2*, *StAR*, *POR*, *SRD5A2*, *CYP11B2*, and *TNXB* using LRS. Firstly, the high-quality DNA is extracted using a blood extraction kit (Meijibio, Guangzhou). The extracted DNA is fragmented using a g-tube (Covaris, United States) and repaired with an end repair enzyme. The repaired DNA is then ligated with Barcode adapters, followed by the purification of the ligation products using Agencourt AMPure XP beads (Beckman Coulter, United States). After the library amplification, the PCR products are purified with Agencourt AMPure XP beads (Beckman Coulter, United States) and quantified with Qubit. Nanodrop is used to assess DNA purity and agarose gel electrophoresis is used to check the degradation and size of DNA fragments. The above products are captured by hybridization using CAH probes, followed by washing and purifying the hybridized products using Agencourt AMPure XP beads (Beckman Coulter, United States). Concentrations are measured using the Qubit dsDNA HS Assay Kit.

Qualified DNA samples are used to construct libraries following the instructions provided with the PacBio Single Molecule Real-Time (SMRT) bellTM Express Template Prep Kit 2.0 (PacBio, United States) kit. The resulting products are purified with Agencourt AMPure XP beads (Beckman Coulter, United States), and the concentrations are determined using the Qubit dsDNA HS Assay Kit. The library fragment sizes are detected using the Agilent 2100 Bioanalyzer (Agilent Technologies, United States). The constructed DNA libraries are then sequenced using the PacBio Sequel II platform (PacBio, United States). After the raw sequencing data is qualified by SMRT Link (version 12.0) to obtain HiFi reads, proceed with subsequent bioinformatics analysis. Circular Consensus Sequence (CCS) reads were automatically generated by the PacBio SMRT analysis module and were mapped to the reference genome GRCh38/hg38 by using Minimap2 (version 2.24). SNVs were called by DeepVariant (version 1.3.0) and annotated by Vep (version 107). SVs were detected by Sniffles (version 2.0.7) and Cutesv (version 1.0.12). SVs were annotated by AnnotSV (version 3.1.1).

#### 2.2.2 MLPA combined with long-PCR sequencing

##### 2.2.2.1 MLPA

Each sample takes 2 mL of blood, and DNA is extracted u sing a blood DNA extraction kit and dissolved in 1 * TE buffer. Using the MLPA kit of MRC-Holland to carry out DNA denaturation, probe hybridization, ligation reaction, amplification and data analysis. 1) Take 50 ng of DNA, add 1*TE buffer to make up to 5 ul, and denature at 98°C for 5 min. 2) After DNA denaturation, add the corresponding probe mixture (1.5 μL MLPA buffer, 1.5 μL probemix), mix well and incubate overnight for 18 h. 3) Add 32 μL ligase mixture (25 μL dH2O, 3 μL Ligase Buffer A, 3 μL Ligase Buffer B, 1 μL Ligase-65 enzyme), mix well and perform ligation reaction. 4) Add PCR mixture (7.5 μL Ultrapure water, 2 μL SALSA PCR primer mix, 0.5 μL SALSA Polymerase), gently mix and perform PCR reaction. 5) The PCR products are detected by ABI 3730XL. 6) The test results are analyzed by COFFALYSER.NET software. COFFALYSER.NET software.

##### 2.2.2.2 Long-PCR

The target gene is amplified by specific PCR amplification primers. The amplification reaction procedure is: 94°C, 5 min; 98°C for 1 min, 68°C for 10 min, 25 cycles; 68°C for 20 min. The PCR product is purified by Ampure magnetic beads and quantified by Qubit. The amplified DNA is broken up by ultrasound, and Ampure magnetic bead fragments are selected to obtain DNA products of 300–400 bp. The second-generation DNA library is constructed using the Rapid Plus DNA Lib Prep Kit for Illumina kit and the Dual DNA Adapter 96 Kit for Illumina kit. The constructed DNA library is subjected to Illumina NovaSeq high-throughput sequencing. After the sequencing data is evaluated to be qualified by Illumina Sequence Control Software (SCS), data reading and bioinformatics analysis are performed. After the sequencing data is evaluated to be qualified by Illumina Sequence Control Software (SCS), data reading and bioinformatics analysis are performed.

### 2.3 *CYP21A2* pathogenic variant types

Based on the different pathogenic variants of the *CYP21A2* gene, it is divided into microconversion events, novel *CYP21A2* variants, deletions (*CYP21A2* Gene Deletion and Chimeric TNXA/TNXB), Chimeric (*CYP21A1P*/*CYP21A2*), and *CYP21A2* Gene Duplications (chimeric *CYP21A2*/*CYP21A1P* chimeric and chimeric TNXB/TNXA (Concolino and Costella, 2018).

#### 2.3.1 Microconversion events

Microconversion refers to the variation formed when the non-functional pseudogene *CYP21A1P* is transferred to the functional gene *CYP21A2* through microconversion events. Microconversion events mainly include: splicing mutations that occur at the end of intron2 (c.293-13A/C > G, also known as I2G); deletion of 8 bp in exon 3 (p.G111Vfs), insertion of 1 nucleotide in exon 7 (p.L308Ffs), a nonsense mutation in exon 8 (p.Q319X), 3 missense mutations (p.P31L, p.I173N and p.R357W), and 1 cluster conversion in exon 6 (p.I237N, p.V238E and p.M240K) (Choi et al., 2016; Concolino and Costella, 2018).

#### 2.3.2 Novel *CYP21A2* variants

Novel *CYP21A2* variants occur without gene conversion, and the mutation sites are not present in *CYPA21A1P*, primarily consisting of private and missense mutations (Pignatelli et al., 2019).

#### 2.3.3 *CYP21A2* gene deletions

The *CYP21A2* gene deletions include complete and partial deletions of the *CYP21A2* gene. Complete absence of the gene can be manifested as either a solo deletion of the *CYP21A2* gene or a chimeric *TNXA*/*TNXB*. The chimeric *TNXA*/*TNXB* results in the absence of the complete *CYP21A2* and part of the *TNXB*, with three most common types being CAH-X CH1 to CH3 (Carrozza et al., 2021). CAH-X CH1 is caused by the complete *CYP21A2* and a 120 bp deletion in the exon 35 of *TNXB*. CAH-X CH2 has a complete exon 35 of *TNXB* but with a complete absence of *CYP21A2* and a p.C4058W mutation in exon 40. CAH-X CH3 has a cluster of three pseudo-genes (p.R4073H in exon 41, p.D4172N and p.S4175N in exon 43) derived mutations in *TNXB* and a complete absence of the *CYP21A2* (Marino et al., 2021). Chimeric *CYP21A1P*/*CYP21A2* gene leads to a partial replacement of the true gene *CYP21A2* with the pseudogene *CYP21A1P*. Nine different chimeric *CYP21A1P*/*CYP21A2* genes have been identified based on their chimeric junctions (Chen et al., 2012). Depending on whether the junction site is upstream or downstream of the I2G mutation, they can be divided into seven chimeric molecules carrying the I2G mutation (CH1, CH2, CH3, CH5, CH6, CH7, and CH8 (Chen et al., 2012; Concolino and Costella, 2018). Two chimeric subjects with *CYP21A1P* promoter and p.P31L mutation (CH4 and CH9) (Chen et al., 2012).

#### 2.3.4 *CYP21A2* gene duplications

The *CYP21A2* gene is generated through genetic recombination during meiosis, producing the RCCX trimeric fragment, which carries one *CYP21A1P* pseudogene copy and two *CYP21A2* genes. It includes the chimeric *CYP21A2*/*CYP21A1P* and the chimeric *TNXB*/*TNXA* (Concolino and Costella, 2018).

### 2.4 Genotype group

The clinical phenotype of 21-OHD is primarily influenced by mutations in alleles that have less impact on enzyme activity (Xu et al., 2013). Based on *in vitro* data of 21-hydroxylase activity in Table 1, *CYP21A2* mutations are categorized from severe to mild as group Null, A, B, and C (Merke and Auchus, 2020; Wedell et al., 1994).

**TABLE 1:** The classification of common *CYP21A2* mutations based on *in vitro* data.

Group	Nul	A	B	C
Phenotype	SW	SW	SV	NC
*In vitro* activity of *CYP21A2* (%)	0	<1	1–2	20–60
Mutations	Deletion	I2G	p.I173N	p.P31L
	Cluster6E			p.P454S
	p.Q319X			p.V282L
	P.R357W			
	p.R484Pfs			
	p.G110fs			
	p.Leu308fs			

*CYP21A2* mutations are categorized from severe to mild as Null, A, B, and C groups.

Cluster6E include: p.I237N, p.V238E, p.M240 mutations.

SW, salt wasting; SV, simple virilizing; NC, non-classic.

The group Null of mutations includes homozygous or compound heterozygous mutations of deletion, p.G110fs, Cluster6E (p.I237N, p.V238E, p.M240K), p.Q319X, p.R357W, or p.R484P. Group A includes homozygous mutations of I2G or compound heterozygous mutations of I2G and group Null. Group B includes homozygous mutations of p.I173N or compound heterozygous mutations of p.I173N and group Null/A. Group C includes homozygous mutations of p.P31L, p.V281L, p.P453S or compound heterozygous mutations with group Null/A/B. Group D is mutations with unclear effects on 21-hydroxylase activity, including homozygous mutations of Group D or compound heterozygous mutations formed between Group D and other groups.

### 2.5 Statistical analysis

The statistical analysis was performed using SPSS 22.1 software. The normality of the data was tested, and the results of non-normally distributed quantitative data were expressed as Mean ± SD, M (P25, P75). Kruskal-Wallis H and Bonferroni tests were used to compare differences among multiple groups. Spearman’s rank correlation analysis was used to determine the relationship between the severity of the disease (SW, SV, NC) and the genotype groups. *P* < 0.05 indicated a statistically significant difference, and r_s_ > 0 indicated a positive correlation.

## 3 Results

### 3.1 The clinical features of 21-OHD patients

A total of 67 patients were analyzed in this study. Among them, 44 were male (65.67%), and 23 were female (34.33%). The male-to-female ratio was approximately 1.9:1, and the age of detection ranged from 0.01 to 11.5 years old, with an average age of 1.7 years old. The patients were divided into three clinical types: SW in 38 cases, SV in 24 cases, and NC in 5 cases. The hormone levels and clinical manifestations of patients in different typing groups are shown in Table 2. The age at initial diagnosis of the SW group was significantly younger than that of the SV and NC groups, and the androstenedione level in the NC group was significantly lower than that in the SW and SV groups, and the testosterone and progesterone levels in the SV group were significantly lower than those in the SW group.

**TABLE 2:** Hormone levels and clinical manifestations at initial diagnosis in 67 patients with 21-OHD.

Clinical presentation	SW (M32/F6)	SV (M9/F15)	NC (M3/F2)	*P*-value	*P*′-value
Age at first diagnosis (year)	0.08 (0.06, 0.17)	3.67 (0.46, 5.15)	3.83 (0.66, 7.46)	<0.05	<0.05^a^
					<0.05^b^
					>0.05^c^
Basic 17-OHP (ng/mL)	63.8 (46.37, 109.16)	67.95 (36.76, 110.13)	36.55 (19.01, 48.49)	>0.05	


AND (ng/mL)	11.85 (2.49, 14.89)	9.28 (4.00, 12.73)	1.56 (0.75, 1.88)	<0.05	>0.05^a^
					<0.05^b^
					<0.05^c^
T (ng/mL)	1.75 (0.67, 5.89)	0.74 (0.51, 1.53)	0.28 (0.23, 0.51)	<0.05	>0.05^a^
					<0.05^b^
					>0.05^c^
P (ng/mL)	14.82 (3.51, 60.00)	6.44 (2.75, 10.61)	0.39 (0.19, 0.95)	<0.05	>0.05^a^
					<0.05^b^
					>0.05^c^
ACTH (pg/mL)	126.8 (102.10, 346.25)	143.00 (82.7, 308.5)	38.2 (29.5, 155.75)	>0.05	


F (nmol/L)	325.28 (158.10, 1047.21)	178.24 (103.11, 278.83)	273.20 (206.74, 560.55)	<0.05	>0.05^a^
					>0.05^b^
					>0.05^c^
Newborn screening Confirmation	5 (M4/F1)	4 (M2/F2)	1 (M1/F0)		
Skin pigmentation	14 (M13/F1)	6 (M1/F3)	1 (M1/F0)		
Ambiguous genitalia	5 (M0/F5)	14 (M0/F14)	2 (M0/F2)		
Premature pubic hair		3 (M3/F0)	1 (M1/F0)		
Growth spurt		13 (M/F)	1 (M1/F0)		
Gastrointestinal symptoms	29 (M26/F3)				

Mean ± SD, M (P25, P75).

M, male; F, female; SW, salt wasting; SV, simple virilizing; NC, non-classic; 17-OHP, 17-hydroxyprogesterone; ACTH, adrenocorticotropic hormone; F, cortisol.

^a^ SW-SV.

^b^ SW-NC.

^c^ SV-NC.

### 3.2 Results of *CYP21A2* gene testing in 67 patients

**FIGURE 1:**
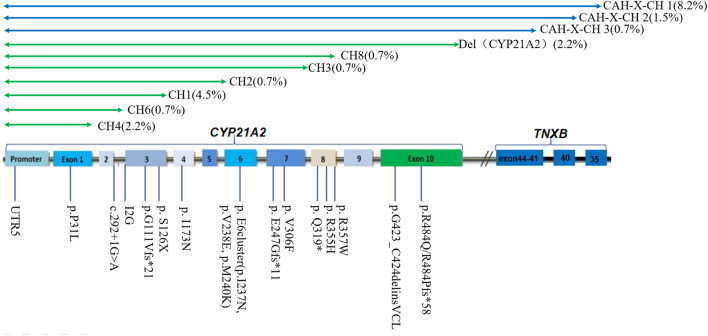
Distribution of *CYP21A2* allelic mutation sites in 67 patients of this study. CH1-9 belongs to chimeric *CYP21A1P*/*CYP21A2*; CAH-X CH1, CAH-X CH2 and CAH-X CH3 belong to chimeric *TNXA*/*TNXB*.

The total number of CYP21A2 allele pathogenic variants detected in 67 patients is 134, while the copy number is 136 (Table 3). Among them, micro-conversions accounted for 61.9%; Novel Variants of *CYP21A2* accounted for 8.2%; deletions accounted for 22.4%, including 12.7% for chimeric *TNXA*/*TNXB*, and 9.7% for chimeric *CYP21A1P*/*CYP21A2*; and *CYP21A2* gene duplications accounted for 3.0%. The results of LRS, MLPA combined with Long-PCR detection, genotype classification, and clinical typing in 67 patients are shown in Table 3. The distribution of mutation sites in the *CYP21A2* alleles of all patients is shown in Figure 1. A total of 29 different mutations were identified in this study. The frequency of *CYP21A2* allele gene mutations are shown in Table 4. The comparison of different detection results between targeted capture LRS and MLPA combined with Long-PCR is shown in Table 5.

**TABLE 3:** Results of LRS and MLPA combined with long-PCR sequencing for *CYP21A2* in 21-OHD.

Patient	Sex	Age	Genotype		LRS	MLPA + Long-PCR	Genotype	Phenotype
		(year)	Allele1	Allele2	Copy 1/Copy 2/Copy 3	(Paternal/Maternal genome)	group	group
1	M	0.08	Microconversion event	TNXA/TNXB	I2G/del (CAH-X-CH2)	I2G/Exon 1-10 Del	A	SW
2	M	0.17	Microconversion event	Novel *CYP21A2* variant	I2G/c.292 + 1G > A	I2G/c.292 + 1G>A	D	SW
3	M	0.08	Microconversion event	CYP21A1P/CYP21A2	p.R357W/UTR5, exon 1-3 (CH1)	p.R357W/Exon 1-3 Del	Null	SW
4	M	0.04	TNXA/TNXB	TNXA/TNXB	del (*CYP21A2*)/del (CAH-X-CH1)	Exon 1-10 Del/**Exon 1-10 Del**	Null	SW
5	M	0.25	Microconversion event	Microconversion event	I2G/I2G	I2G/I2G	A	SW
6	M	0.06	Novel *CYP21A2* variant	CYP21A1P/CYP21A2	p.E247Gfs*11/UTR5, exon 1-5 (CH2)	p.E247Gfs*11/**Exon 1-4 Del**	D	SW
7	F	1.33	Microconversion event	TNXA/TNXB	p.I173N/del (CAH-X-CH3)	p.I173N/Exon 1-10 Del	B	SV
8	F	0.08	CYP21A2/CYP21A1P	CYP21A1P/CYP21A2	Exon 7-8 integration/exon 7-8 integration/UTR5, exon 1 (CH4)/Exon 7-8 integration (p.V282L, p.L308Ffs*6, p.Q319X)	p.L308Ffs*, p.Q319X/p.P31L	C	SV
9	M	0.05	CYP21A1P/CYP21A2	TNXA/TNXB	UTR5, exon 1-8 (CH8)/del (CAH-X-CH1)	Exon 1-10 Del/Exon 1-10 Del	Null	SW
10	M	0.04	Microconversion event	CYP21A1P/CYP21A2	I2G/UTR5, part of exon 1-8 (CH3)	I2G/Exon 1-7 Del	A	SW
11	M	0.08	Microconversion event	TNXA/TNXB	I2G/del (CAH-X-CH2)	I2G/Exon 1-10 Del	A	SW
12	M	0.08	CYP21A2/CYP21A1P	CYP21A1P/CYP21A2	Exon 7-8 integration/exon 7-8 integration/UTR5, exon 1-3 (CH1)/Exon 7-8 integration (p.V282L, p.L308Ffs*6, p.Q319X)	p.L308Ffs*, p.Q319X/Exon 1-3 Del	C	SW
13	M	5.25	Microconversion event	CYP21A1P/CYP21A2	p.I173N/UTR5, part of exon 1-3, including IG2, excluding del 8 bp (CH6)	p.I173N/**I2G**	B	SV
14	M	8.33	Microconversion event	TNXA/TNXB	p.P31L/del (*CYP21A2*)	p.P31L/Exon 1-10 Del	C	NC
15	F	3.08	Microconversion event	CYP21A1P/CYP21A2	I2G/UTR5, exon 1-3 (CH1)	I2G/Exon 1-3 Del	A	SV
16	F	0.17	Microconversion event	TNXA/TNXB	I2G/del (*CYP21A2*)	I2G/Exon 1-10 Del	A	SW
17	M	0.06	Microconversion event	Microconversion event	I2G/p.R357W	I2G/p.R357W	A	SW
18	F	0.06	Microconversion event	Microconversion event	I2G/I2G	I2G/I2G	A	SW
19	M	0.58	Microconversion event	Microconversion event	I2G/I2G	I2G/I2G	A	SW
20	M	0.04	Microconversion event	Microconversion event	p.I173N/p.I173N	p.I173N/p.I173N	B	SW
21	M	4.83	Microconversion event	Microconversion event	I2G/p.I173N	I2G/p.I173N	B	SV
22	M	0.17	Microconversion event	Novel *CYP21A2* variant	R357W/p.R484Pfs*58	p.R357W/p.R484Pfs	Null	SW
23	M	0.08	Microconversion event	Novel *CYP21A2* variant	I2G/p.S126X	I2G/p.S126X	D	SW
24	F	3.92	Microconversion event	CYP21A1P/CYP21A2	p.I173N/UTR5, exon 1-3(CH1)	p.I173N/Exon 1-3 Del	B	SV
25	F	4.08	Microconversion event	Microconversion event	I2G/p.I173N	I2G/p.I173N	B	SV
26	F	0.08	Microconversion event	Novel *CYP21A2* variant	p.I173N/p.V306F	p.I173N/p.V306F	D	SV
27	M	11.5	Microconversion event	TNXA/TNXB	p.I173N/del (CAH-X-CH1)	p.I173N/Exon 1-10 Del	B	SV
28	F	5.33	Microconversion event	Novel *CYP21A2* variant	p.P31L/R484Pfs*58	p.P31L/p.R484Pfs	C	SV
29	M	0.17	Microconversion event	CYP21A1P/CYP21A2	p.R357W/UTR5, exon 1-3 (CH1)	p.R357W/Exon 1-3 Del	Null	SW
30	M	0.5	Microconversion event	Novel *CYP21A2* variant	I2G/p.G423_C424delinsVCL	I2G/p.G423_C424delinsVCL	D	SW
31	F	4.67	Microconversion event	Novel *CYP21A2* variant	p.R484Pfs*58/p.I173N	*In vitro* fertilization from sperm bank/p.I173N	B	SV
32	M	0.05	Microconversion event	TNXA/TNXB	p.Q319X/del (CAH-X-CH1)	p.Q319X/Exon 1-10 Del	Null	SW
33	F	2.91	Microconversion event	Microconversion event	I2G/p.I173N	I2G/p.I173N	B	SV
34	M	3.33	Microconversion event	Novel *CYP21A2* variant	p.I173N/c.292 + 1G > A	p.I173N/c.292 + 1G > A	D	SW
35	M	0.17	Microconversion event	Novel *CYP21A2* variant	I2G/p.R355H	I2G/p.R355H	D	SW
36	M	0.07	Microconversion event	Microconversion event	I2G/p.I173N	I2G/p.I173N	B	SV
37	F	3.17	Microconversion event	Microconversion event	p.G111Vfs*21/p.I173N	p.G111Vfs*21/p.I173N	B	SV
38	M	0.08	Microconversion event	Novel *CYP21A2* variant	I2G/p.S126X	I2G/p.S126X	D	SW
39	M	0.32	Microconversion event	Novel *CYP21A2* variant	p.I173N/p.R484Pfs*58	p.I173N/p.R484Pfs	B	SW
40	M	0.08	Microconversion event	Microconversion event	I2G, p.G111Vfs*21/I2G	I2G, p.G111Vfs*21/I2G	A	SW
41	M	3.66	Microconversion event	Novel *CYP21A2* variant	p.I173N/c.292 + 1G > A	p.I173N/c.292 + 1G > A	D	SV
42	F	0.17	Microconversion event	Microconversion event	I2G/p.I173N	I2G/p.I173N	B	SW
43	M	0.17	Microconversion event	Microconversion event	I2G/p.R357W	I2G/p.R357W	A	SW
44	F	2.42	Microconversion event	Microconversion event	p.I173N/p.Q319X	p.I173N/p.Q319X	B	SV
45	F	1.17	CYP21A1P/CYP21A2	CYP21A2/CYP21A1P	**UTR5, exon 1 (CH4)**/p.L308Ffs*6, p.Q319X	**p.P31L**/p.L308Ffs*6, p.Q319X	C	NC
46	F	3.83	CYP21A1P/CYP21A2	CYP21A2/CYP21A1P	**UTR5, exon 1 (CH4)**/p.L308Ffs*6, p.Q319X	**p.P31L**/p.L308Ffs*6, p.Q319X	C	NC
47	M	0.03	Microconversion event	Microconversion event	p.Q319X/p.R357W	p.Q319X/p.R357W	Null	SW
48	M	0.04	Microconversion event	Microconversion event	I2G/I2G	I2G/I2G	A	SW
49	F	6.17	Microconversion event	TNXA/TNXB	I2G/del (CAH-X-CH1)	I2G/Exon 1-10 Del	A	SV
50	F	0.08	Microconversion event	Microconversion event	p.I173N/p.I173N	p.I173N/p.I173N	B	SV
51	F	0.08	TNXA/TNXB	TNXA/TNXB	del (CAH-X-CH1)/del (CAH-X-CH1)	Exon 1-10 Del/Exon 1-10 Del	Null	SW
52	M	3.67	Microconversion event	Novel *CYP21A2* variant	p.I173N/p.S126X	p.I173N/p.S126X	D	SV
53	M	0.32	Microconversion event	TNXA/TNXB	I2G/del (CAH-X-CH1)	I2G/Exon 1-10 Del	A	SW
54	M	0.17	Microconversion event	Novel *CYP21A2* variant	I2G/p.R484Pfs*58	I2G/p.R484Pfs	A	SW
55	F	0.01	Microconversion event	Microconversion event	p.Q319X/p.R357W	p.Q319X/p.R357W	Null	SV
56	M	0.17	Microconversion event	Microconversion event	E6cluster/p.R357W	E6cluster/p.R357W	Null	SV
57	F	0.02	Microconversion event	TNXA/TNXB	I2G/del (CAH-X-CH1)	I2G/Exon 1-10 Del	A	SW
58	M	0.65	Microconversion event	Microconversion event	UTR5, p.Q319X/**p.R357W**	p.Q319X/**negative**	Null	SW
59	M	0.05	CYP21A1P/CYP21A2	TNXA/TNXB	del (CH1)/del (CAH-X-CH1)	Exon 1-3 Del/Exon 1-10 Del	Null	SW
60	M	4.25	Microconversion event	Microconversion event	I2G/p.I173N	I2G/p.I173N	B	SV
61	M	6.58	Microconversion event	Microconversion event	p.P31L/E6cluster	p.P31L/E6cluster	C	NC
62	M	0.15	Microconversion event	TNXA/TNXB	p.P31L/del (CAH-X-CH1)	p.P31L/Exon 1-10 Del	C	NC
63	M	6.67	Microconversion event	Microconversion event	I2G/p.I173N	I2G/p.I173N	B	SV
64	M	0.12	Microconversion event	Microconversion event	I2G/Q319X	I2G/p.Q319X	A	SW
65	F	7.33	Microconversion event	Novel *CYP21A2* variant	I2G/R484Q	I2G/p.R484Q	D	SV
66	M	0.23	Microconversion event	Novel *CYP21A2* variant	I2G/R484Pfs*58	I2G/p.R484Pfs	A	SW
67	F	0.17	Microconversion event	Microconversion event	I2G/R357W	I2G/R357W	A	SW

M, male; F, female.

The parts in bold black are the different detection results of targeted capture and LRS combined with MLPA and Long-PCR.

**TABLE 4:** Mutation frequency of *CYP21A2* allele in this study.

Allelic mutation		Frequency	Rate
Microconversion events	c.293–13A/C > G (I2G)	36	26.9%
	p.G111Vfs*21	1	0.7%
	p.Q319X	5	3.7%
	p.P31L	4	3.0%
	p.I173N	23	17.2%
	p.R357W	10	7.5%
	E6 cluster	2	1.5%
	UTR5 (−103, −110, −113, −126), p.Q319X	1	0.7%
	I2G, p.G111Vfs*21	1	0.7%
*CYP21A2* Gene deletion	*TNXA/TNXB*		
	CAH-X-CH 1	11	8.2%
	CAH-X-CH 2	2	1.5%
	CAH-X-CH 3	1	0.7%
	*CYP21A1P*/*CYP21A2*		
	Del (*CYP21A2*)	3	2.2%
	CH1	6	4.5%
	CH2	1	0.7%
	CH3	1	0.7%
	CH4	3	2.2%
	CH6	1	0.7%
	CH8	1	0.7%
*CYP21A2* Gene duplication	*TNXB/TNXA*	0	0.0%
	*CYP21A2/CYP21A1P*	4	3.0%
Novel *CYP21A2* variants	R484Pfs*58	6	4.5%
	R484Q	1	0.7%
	c.292 + 1G > A	3	2.2%
	p.S126X	3	2.2%
	p.E247Gfs*11	1	0.7%
	p.V306F	1	0.7%
	p.G423 C424delinsVCL	1	0.7%
	p.R355	1	0.7%

E6 cluster include: p.I237N, p.V238E and p.M240K.

**TABLE 5:** The comparison of different detection results between LRS and MLPA combined with Long-PCR.

LRS	MLPA + Long-PCR
chimeric *TNXA*/*TNXB*	
CAH-X-CH1	Exon 1-10 Del
	Exon 1-10 Del
	Exon 1-10 Del
	Exon 1-10 Del
	Exon 1-10 Del
	Exon 1-10 Del
	Exon 1-10 Del
	Exon 1-10 Del
	Exon 1-10 Del
	Exon 1-10 Del
	Exon 1, 3, 4, 6, 7 Del
CAH-X-CH2	Exon 1-10 Del
	Exon 1-10 Del
CAH-X-CH3	Exon 1-10 Del
chimeric CYP21A1P/CYP21A2	
CH1 (UTR5, exon 1-3)	Exon 1-3 Del
	Exon 1-3 Del
	Exon 1-3 Del
	Exon 1-3 Del
	Exon 1-3 Del
CH2 (UTR5, exon 1-5)	Exon 1-4 Del
CH3 (UTR5, part of exon 1-8)	Exon 1-7 Del
CH4 (UTR5, exon 1)	p.P31L
	p.P31L
	p.P31L
CH6(UTR5, part of exon 1-3, including IG2, excluding del 8 bp)	I2G
CH8(UTR5, exon 1-8)	Exon 1-10 Del
CYP21A2/CYP21A1P	
[exon 7-8 integration (V282L, L308Ffs*6, p.Q319X)]	p.L308Ffs*, p.Q319X
	p.L308Ffs*, p.Q319X
p.R357W	—
p.R484Pfs*58	Sperm Bank IVF

The overall genotype-phenotype correlation was 82.1%, with a positive predictive rate of 83.3% of the Null group in SW, 89.47% of Group A in SW, 82.4% of Group B in SV, and 62.5% of Group C in NC. The correlation coefficient r_s_ between the severity of the phenotype and the genotype grouping was 0.682 (*P* < 0.05) (Table 6).

**TABLE 6:** Relationship between genotype and clinical phenotype.

Group	SW	SV	NC
Null	10	2	0
A	17	2	0
B	3	14	0
C	1	2	5

SW, salt wasting type; SV, simple virilization type; NC, nonclassical type.

#### 3.2.1 Microconversion events

In this cohort, 83 variants were derived from the transformation of the *CYP21A1P* pseudogene, with mutation frequencies as follows: I2G (36/134, 26.9%), p.I173N (23/134, 17.2%), p.R357W (10/134, 7.5%), p.Q319X (5/134, 3.7%), p.P31L (4/134, 3.0%), p.G111Vfs*21 (1/134, 0.7%), E6 cluster (p.I237N, p.V238E, and p.M240K) (2/134, 1.5%), UTR5 and p.Q319X (1/134, 0.7%), I2G and p.G111Vfs*21 (1/134, 0.7%).

#### 3.2.2 Novel *CYP21A2* variants

Novel *CYP21A2* variants were detected in 17 alleles. Seven rare variants of *CYP21A2* include R484Pfs*58, c.292 + 1G > A, S126X, E247Gfs*11, V306F, p.G423_C424delinsVCL (c.1268_1275delinsTGTGCCTGGGC), and R355H. Notably, p.G423_C424delinsVCL (c.1268_1275delinsTGTGCCTGGGC) is a newly discovered mutation in this study and has not been reported in the HGMD pro database.

#### 3.2.3 *CYP21A2* gene deletions

A total of 30 allelic mutations were identified in the patients in this cohort. Among them, 17 were complete losses of *CYP21A2*, and 13 were partial losses. These included chimeric *TNXA*/*TNXB* CAH-X-CH1 accounted for 8.2% (11/134), CAH-X-CH2 accounted for 1.5% (2/134), CAH-X-CH3 accounted for 0.7% (1/134). In chimeric *CYP21A1P*/*CYP21A2*, deletion (Del) (*CYP21A2)* accounted for 2.2% (3/134), followed by CH1 accounted for 4.5% (6/134), CH4 accounted for 2.2% (3/134), and CH2 0.7% (1/134), CH3 0.7% (1/134), CH6 0.7% (1/134), and CH8 0.7% (1/134), respectively.

LRS detected the chimeric *TNXA*/*TNXB* CAH-X-CH1 mutation, and the results of second-generation sequencing combined with MLPA showed Exon 1, 3, 4, 6, 7 Del or Exon 1-10 Del; for the CAH-X-CH2 mutation, the results showed Exon 3 Del; for the CAH-X-CH3 mutation, the results showed Exon 1-10 Del. For the chimeric *CYP21A1P*/*CYP21A2* CH1 mutation, the results showed Exon 1-3 Del or p.P31L; the CH2 showed Exon 1-5 Del; the CH3 showed Exon 1-6 Del; the CH4 showed p.P31L, but UTR5 was not detected; the CH6 showed I2G, and UTR5 and Exon 1-3 partial Del were not detected. The results above were detected using a combination of second-generation sequencing and MLPA.

#### 3.2.4 *CYP21A2* gene duplications

Gene recombination during meiosis can lead to duplication of the *CYP21A2* gene, including *CYP21A2*/*CYP21A1P* chimeras and *TNXB*/*TNXA* chimeras. In this cohort of patients, a total of four *CYP21A2* gene duplications were detected, all of which were chimeric *CYP21A2*/*CYP21A1P*, with no chimeric *TNXB*/*TNXA* identified. The combined results of next-generation sequencing and MLPA for patients 08 and 12 showed p.L308Ffs*, p.Q319X mutations, but no p.V282L mutation was detected.

## 4 Discussion

21-OHD has high clinical and genetic heterogeneity and seriously impacts the quality of life for patients. Neonatal screening for 21-OHD has been implemented in some regions, but traditional screenings based on 17-hydroxyprogesterone (17-OHP) concentrations have a certain false-positive rate, limiting their diagnostic value (Guran et al., 2020; Lind-Holst et al., 2022; Tippabathani et al., 2023; White, 2009). Currently, genetic testing is recommended as a secondary screening tool for 21-OHD (Merke and Auchus, 2020). Genetic testing technology for 21-OHD has progressed from Sanger sequencing combined with QPCR, and NGS combined with MLPA, to LRS. LRS can obtain target genes through either specific long-range PCR amplification or probe capture methods. The LRS based on specific long PCR amplification has been reported to detect five genes: *CYP21A2*, *CYP11B1*, *CYP17A1*, *HSD3B2*, and *StAR*, and can amplify highly homologous sequences that are difficult to amplify by conventional PCR. The length of the single amplification can reach 5–20 kb. However, long-range PCR amplification is mainly used for prenatal screening and can only detect common variants that have been reported (Li et al., 2023; Liu et al., 2022; Zhang et al., 2023). In this study, LRS used probe capture to achieve the capture and to enrich target genes. The method can detect ten genes: *CYP21A2*, *CYP11B1*, *CYP17A1*, *HSD3B2*, *StAR*, *CYP11A1*, *POR*, *CYP11B2*, *SRD5A2*, and *TNXB*, with a wider range of detection that is not limited to previously reported variations. Compared with Sanger and NGS sequencing methods, LRS can accurately distinguish between true genes and pseudogenes, tandem repeats, and directly detect structural variants, such as microrearrangements of *CYP21A2*, *CAH-X* mutations from CH1 to CH3, *CYP21A1P/CYP21A2* chimeras (CH1-CH9), *CYP21A2/CYP21A1P* chimeras, and *TNXB*/*TNX* chimeras, etc. In addition, the method can accurately determine the cis/trans configurations of the variants without parental gene validation.

This study used targeted capture LRS, MLPA combined with Long-PCR to detect *CYP21A2* mutations. There were 30 differences in the results of the detection. Patient 31 was conceived through *in vitro* fertilization (IVF) using donor sperm from a sperm bank. The MLPA combined with Long-PCR could not determine the origin of his mutation. LRS could identify the cis-trans configuration of patients without family verification, helping patients with genetic diagnosis. MLPA combined with Long-PCR did not detect the p.R357W mutation in patient 58, which was inherited from the father, but LRS did, aiding in identifying the pathogenic gene. LRS detected the integration of exon 7-8 including p.V282L (exon 7), p.L308Ffs * (exon 7), p.Q319X (exon 8) mutations in patients 8 and 12, while MLPA combined with Long-PCR failed to detect p.V282L. This may be related to Long-PCR’s tendency to off-target.

Additionally, *CYP21A2* is highly homologous to *CYP21A1P*, with only 65 nucleotide differences between the active gene *CYP21A2* and the pseudogene *CYP21A1P* across the exons and introns regions (White et al., 1986). If NGS sequencing is used, the resulting gene fragments are only about 200 bp in size. The high similarity between the target and the homologous regions can lead to ambiguous matching of the reference sequence, making it difficult to fully distinguish between the true gene and pseudogenes. At the same time, the specific probes used by MLPA are also difficult to detect fixed complex rearrangements. Furthermore, LRS can detect the specific variations in each copy. Patients 08 and 12 have three copy number variations with exon 7-8 fusion. The fusion of exon 7-8 is one type of chimeric *CYP21A2/CYP21A1P*, with a carriage rate of approximately 7%. This is not a complete duplication of *CYP21A2*, but rather a partial duplication of certain exons within the *CYP21A2* gene (Parajes et al., 2008). p.Q319X is usually associated with gene duplication of *CYP21A2* and a functional loss of *CYP21A2* (Kleinle et al., 2009). This study identified a total of 14 chimeric *TNXA*/*TNXB*, but the results of MLPA combined with Long-PCR detection were all lack of exon 1-10 of *CYP21A2*. This indicates that CAH-X-CH1, CAH-X-CH2 and CAH-X-CH3 cannot be distinguished from each other. This is related to the probe sets in CAH-MLPA which are composed of four areas. Area 1 contains four probes of *CYP21A1P*, area 2 contains eight probes of *CYP21A2* exon 1-7, area 3 contains six probes of *TNXB*, and area 4 is a reference probe. The six probes of *TNXB* include two sites on exon 35 and one site on exon 19, 20, 29 and 31. Therefore, it cannot detect deletions in specific exons from *TNXB* exon 40-44. In other words, it cannot distinguish different structural variations from CAH-X-CH1 to CAH-X-CH3 in chimeric *TNXA/TNXB*. However, the BAM figure of LRS can clearly show the fusion situation of active gene *CYP21A2* and the pseudogene *CYP21A1P* that occurred in patients. This study identified 12 instances of chimeric *CYP21A1P*/*CYP21A2*. The LRS detected recombinations in CH1 (UTR5, exon 1-3), CH2 (UTR5, exon 1-5), CH3 (UTR5, part of exon 1-8), CH4 (UTR5, exon 1), CH6 (UTR5, part of exon 1-3, including IG2, excluding del 8 bp), and CH8 (UTR5, exon 1-8). The results of combined MLPA and Long-PCR were Exon 1-3 Del, Exon 1-4 Del, Exon 1-7 Del, p.P31L, I2G, and Exon 1-10 Del. The discrepancies in the detection results were due to the limited probe coverage of *CYP21A2* in region 2 by MLPA, which only contained probes for exons 1, 3, 4, 6, 7, and I2G. In CH1, the mutation in exon 1-3 detected by combined MLPA combined with Long-PCR was inferred to be a deletion of exon 1-3 based on the detection of missing *CYP21A2* probes for exon 1 and exon 3. In CH2, MLPA combined with Long-PCR failed to detect a mutation in exon 5 because no probe was set for it, thus the deletion of exon 5 could not be detected. Similarly, in CH3, MLPA combined with Long-PCR failed to detect a partial deletion in exon 8 due to the absence of a probe for *CYP21A2* exon 8. The deletion in exon 1 of CH4 was consistent with the detection of p.P31L by MLPA combined with Long-PCR. In CH6, the failure of MLPA combined with Long-PCR to completely distinguish between true and false genes led to the undetected mutation in exon 1. In CH8, MLPA combined with Long-PCR, which did not include probes for *CYP21A2* exons 8, 9, and 10, failed to detect deletions in these exons. The reported Exon 1-10 Del result was merely an inference based on the absence of exons 1, 3, 4, 6, 7.

This study found a concordance rate of 82.1% (46/56 cases) between genotype and phenotype, which is generally similar to other reports (Finkielstain et al., 2011; New et al., 2013; Riedl et al., 2019; Stikkelbroeck et al., 2003). The mutations in Group Null and Group A affect the key functions of 21-hydroxylase, causing alterations in membrane anchoring, heme binding, or enzyme stability, leading to the complete loss of 21-hydroxylase function. The mutations in Group B affect the transmembrane regions or conserved hydrophobic patches, managing to retain only 1%–2% of the normal enzyme activity (Higashi et al., 1988; Tusie-Luna et al., 1990). The mutation in Group C disrupts the interaction of redox enzymes, salt bridges, and hydrogen bond networks, retaining 20%–60% activity of 21-hydroxylase (Helmberg et al., 1992; Tusie-Luna et al., 1991; Tusie-Luna and White, 1995). Previous studies have shown a stronger correlation between genotype and phenotype for group Null and A mutations, while patients with group B and C mutations exhibit greater genotype-phenotype variability (Riedl et al., 2019). The genotype-phenotype variability rate in group B patients is 17.6%, which may be associated with alterations in transcriptional regulation of the protein or downstream protein translation (New et al., 2013). The positive prediction rate of group C for NC is only 62.5%. Overall, the p.V283L and p.P454S mutation genotypes in group C have a relatively good correlation with the phenotype, while the genotype-phenotype variation for the p.P31L genotype is greater (Krone et al., 2000; New et al., 2013). The sequencing results of patients 08 and 12 in group C of this study showed the p.P31L mutation, but their corresponding LRS results were actually CH4 and CH1, respectively. In patient 08, the chimeric *CYP21A1P*/*CYP21A2* CH4 had a deletion at a site upstream of the I2G mutation. CH4 carried two mutations: the *CYP21A1P* promoter and p.P31L, which had a relatively weak impact on the activity of 21-hydroxylase, presenting with the clinical phenotype of SV. In patient 12, the deletion site of CH1 was downstream of the I2G mutation, containing I2G and multiple pseudogene mutations. This mutation had a greater impact on the 21-hydroxylase, with the clinical phenotype being SW. The LRS test results can better assist in the clinical typing of 21-OHD. Of course, the genotype-phenotype variation in 21-OHD patients is caused by various factors. In addition to *CYP21A2* mutations, the length of the CAG repeats in the androgen receptor (Kaupert et al., 2013; Moura-Massari et al., 2016), the high polymorphism of the protein P450 oxidoreductase, and the splicing mutations in RNA (Buchner et al., 2003; L’Allemand et al., 2000), can all affect the patient’s phenotype. Genotype-phenotype inconsistencies require further study.

Approximately 95% of disease-causing variants arise from microconversions during meiosis, unequal crossing-over, deletions, and the formation of non-functional chimeric genes in the *CYP21A2* gene (Merke and Auchus, 2020; Werkmeister et al., 1986). In this study, targeted capture and LRS on the PacBio Sequel II platform were employed to detect 28 types of *CYP21A2* pathogenic mutations in 67 21-OHD patients. Microconversions accounted for 61.9%, novel *CYP21A2* variants for 8.2%, deletions for 22.4%, and *CYP21A2* gene duplication for 3.0%. Microconversions, chimeric *TNXA*/TNX, chimeric *CYP21A1P*/*CYP21A2*, and chimeric *CYP21A2*/*CYP21A1P* mutations altogether accounted for 91.8% of the mutations. This proportion is roughly similar to other reported studies. The I2G mutation had the highest proportion (26.9%), followed by p.I173N (17.2%) and CAH-X-CH1 (8.2%). I2G is currently the most common mutation affecting splicing, located in the most polymorphic region of the *CYP21A2* gene (Concolino and Costella, 2018). I2G is also the most common in Western Alaska Eskimos and Iranians (Wilson et al., 2007). No suspicious point mutations were detected in exons 2, 5, and 9 during this test, which may be related to the fact that these regions contain non-differential sequences of pseudo-genes and are less likely to undergo recombination or conversion.

Although LRS has longer sequencing read length and higher accuracy, it also has limitations. LRS has higher requirements for sample quality, and the sample cellular DNA samples are prone to degradation. In addition, the price of the LRS sequencer is high, and the single-molecule sequencing chip is not reusable, and the reagent price is relatively expensive. At the same time, the HiFi sequencing obtains Circular Consensus Sequencing (CCS) sequences, which can greatly improve the accuracy of the test, but at the same time, the amount of data generated after the test is large, and bioinformatics analysis requires a lot of manpower and computing resources, resulting in high costs.

The targeted capture based on the PacBio Sequel II platform and LRS detection technology will become an important tool for molecular diagnosis in the near future. LRS detection can achieve precise genotyping of candidate genes for CAH in one step, including microtransformation, new variations, deletions, and duplications. It can determine the trans/cis position of the variation without detecting the *CYP21A2* gene of the patient’s parents. However, the sample size of this study is relatively limited, and the advantages of other types of CAH have not been fully demonstrated. Large-scale, multicenter prospective studies are still needed to maximize the advantages of LRS.

## Data Availability

The data presented in the study are deposited in the NCBI repository, accession number PRJNA1152331.
