# Supplementary figures and images for: Comparison of long-read sequencing and MLPA combined with long-PCR sequencing of CYP21A2 mutations in patients with 21-OHD
Source: Front Genet. 2024 Nov 1;15:1472516. doi: 10.3389/fgene.2024.1472516 (PMC11563783; doi:10.3389/fgene.2024.1472516)

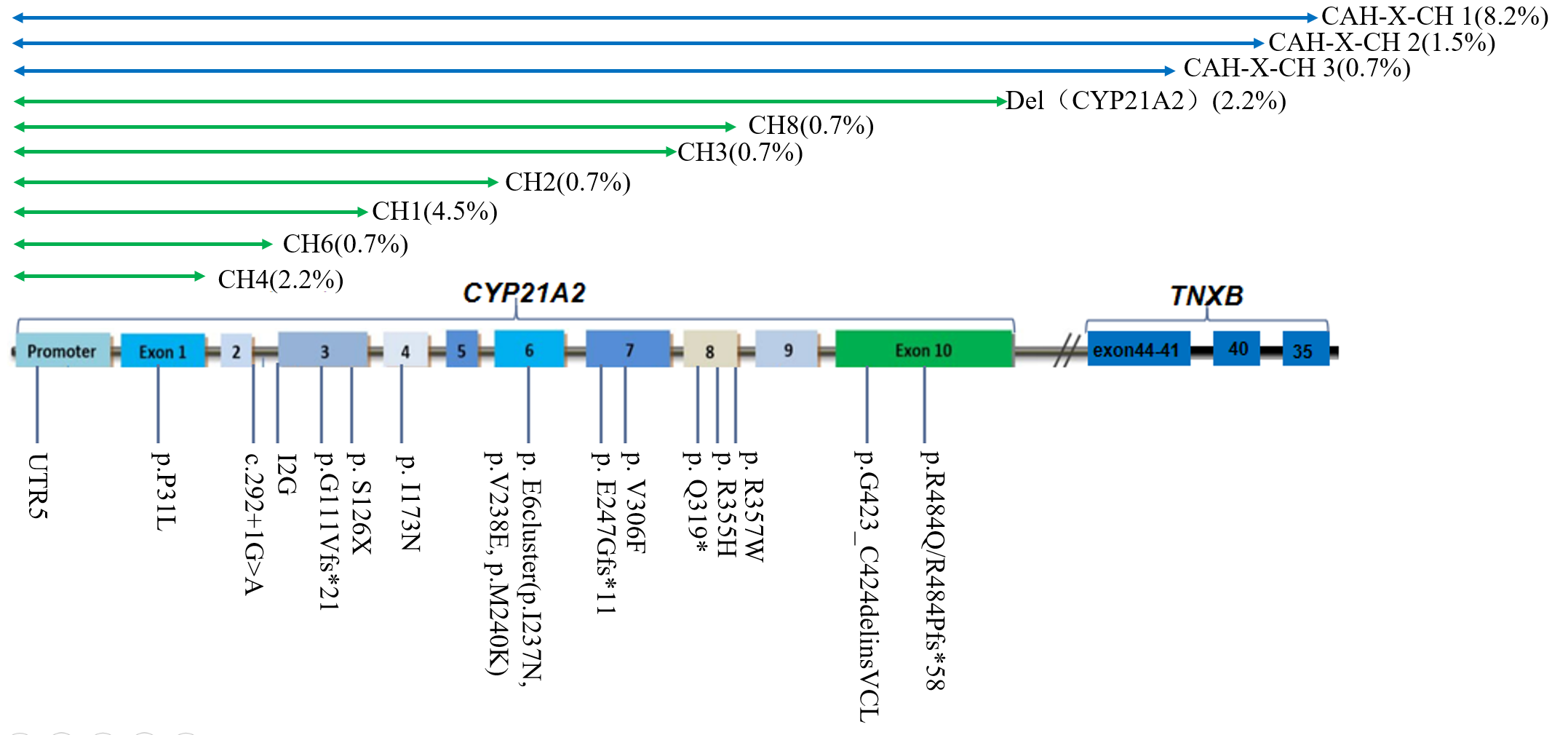

Supplement: Supplementary file 1 [file Image1.PNG]
